# A Comparative Analysis of AI Models in Complex Medical Decision-Making Scenarios: Evaluating ChatGPT, Claude AI, Bard, and Perplexity

**DOI:** 10.7759/cureus.52485

**Published:** 2024-01-18

**Authors:** Vamsi Krishna Uppalapati, Deb Sanjay Nag

**Affiliations:** 1 Department of Anesthesiology, Tata Main Hospital, Jamshedpur, IND

**Keywords:** future medicine, ai efficacy, ai comparison, healthcare ai, medical decision-making

## Abstract

This study rigorously evaluates the performance of four artificial intelligence (AI) language models - ChatGPT, Claude AI, Google Bard, and Perplexity AI - across four key metrics: accuracy, relevance, clarity, and completeness. We used a strong mix of research methods, getting opinions from 14 scenarios. This helped us make sure our findings were accurate and dependable. The study showed that Claude AI performs better than others because it gives complete responses. Its average score was 3.64 for relevance and 3.43 for completeness compared to other AI tools. ChatGPT always did well, and Google Bard had unclear responses, which varied greatly, making it difficult to understand it, so there was no consistency in Google Bard. These results give important information about what AI language models are doing well or not for medical suggestions. They help us use them better, telling us how to improve future tech changes that use AI. The study shows that AI abilities match complex medical scenarios.

## Introduction

In the modern era of digital healthcare, artificial intelligence (AI) has emerged as a pivotal force in transforming medical decision-making [1]. The ability of AI to analyze vast datasets, recognize patterns, and generate predictive models has led to more informed and efficient healthcare delivery [2]. While the adoption of AI in medicine is promising, it introduces a complex landscape of diverse AI models, each with unique capabilities and limitations. Models such as ChatGPT, Claude AI, Bard, and Perplexity have shown the potential to provide medical guidance [3]. However, the healthcare sector necessitates critically evaluating these models to ensure the advice’s accuracy, reliability, and appropriateness.

This study compares and evaluates the performance of AI models regarding medical guidance. The primary objectives include assessing the accuracy of medical information, adherence to current medical guidelines, and the models’ ability to handle complex medical scenarios.

## Technical report

The scope of this research encompasses a systematic examination of the AI tools, including ChatGPT-4, Claude AI (Pro), Google Bard (Pro), and Perplexity (Pro) models, across a spectrum of medical scenarios. In this research, results from four top AI smart systems were assessed. These scenarios were carefully chosen to represent a range of medical conditions and decision-making scenarios, from emergency procedures to chronic disease management.

Every answer AI gave was checked against the gold standards to follow, representing the highest level of agreement in medicine and evidence-based guidelines. The scoring was conducted on dimensions: accuracy, dependability, relevance, and completeness. The AI's diagnosis or treatment advice was checked to match the standard protocol. The answers from AI tools were compared not only for correctness but also for completeness. Reliability was checked by looking at how constantly, trustworthy, and clear the AI's thought process was. A group of doctors from diverse departments like Anaesthesia, Emergency Care, Critical Care, and Cardiology with experience of more than 10 years, who are knowledgeable about medicine and the gold standard medical procedures were used to give the scores from 1 (bad) to 5 (good) based on the Likert scale [4]. This two-part scoring system was made to look at how each AI tool works. The following statistical tests such as descriptive statistics (mean and standard deviations), analysis of variance (ANOVA), and correlations analysis are applied using Jeffreys's Amazing Statistics Program (JASP) (University of Amsterdam, Amsterdam, The Netherlands) to compare how well the AI models present the accuracy and dependability [5].

Medical scenarios and scores

Table 1 presents 14 complex medical scenarios searched in AI tools for suggestions. Each was carefully rated using four AI models to check the accuracy, relevance, clarity, and completeness. The scores showed big differences between the models. Claude AI consistently got higher relevance and completeness marks, while Google Bard's clarity scores were greatly low. Each model had varying user experience quality ratings shown in their test results.

**Table 1:** Evaluation of AI Performance in Medical Scenario Analysis: Assessing ChatGPT, Claude AI, Google Bard, and Perplexity AI

Scenario	ChatGPT Accuracy	ChatGPT Relevance	ChatGPT Clarity	ChatGPT Completeness	Claude AI Accuracy	Claude AI Relevance	Claude AI Clarity	Claude AI Completeness	Google Bard Accuracy	Google Bard Relevance	Google Bard Clarity	Google Bard Completeness	Perplexity AI Accuracy	Perplexity AI Relevance	Perplexity AI Clarity	Perplexity AI Completeness
What is the protocol for intraoperative aneurysm clipping accidental rupture intraoperative? [6]	5	1	4	3	2	5	1	3	3	1	1	2	4	4	1	3
Penetrating eye injury scheduled for emergency laparotomy scoline as muscle relaxant of choice [7]	1	2	4	1	2	5	1	4	4	4	4	4	4	3	3	5
Indication for tracheostomy in context of cancer (ca) buccal mucosa for composite resection surgery [8]	4	2	4	1	4	4	2	3	2	3	1	1	2	4	4	4
Complete tracheal injury role of cricothyrotomy for securing the airway [9]	4	1	1	5	4	5	3	4	3	4	3	1	1	3	4	2
Atrial switch surgery prognosis [10]	4	2	2	1	3	5	5	3	2	1	3	1	1	5	2	3
Malignancy periampullary pancreas post-operative success rate [11]	2	5	2	5	4	5	3	2	5	5	1	2	2	4	4	4
Fat embolism in polytrauma role of emergency pelvic surgery in emergency [12]	4	4	2	2	1	1	1	3	3	1	5	1	3	4	5	2
Open prostate surgery in 250 gm prostate grade prognosis [13]	3	1	1	5	4	5	4	4	4	1	4	5	1	1	4	5
Elective ventilation's role in a post-COVID patient with bilateral basal atelectasis and interstitial lung disease scheduled for laparoscopic cholecystectomy [14]	5	4	3	2	5	4	3	4	1	3	5	2	4	4	4	3
Tetralogy of Fallot child scheduled for emergency rigid bronchoscopy for foreign body removal anesthesia concerns [15]	1	1	5	3	2	3	3	4	4	4	1	4	2	1	2	3
Peritonsillar abscess with difficult airway, full stomach, stridor for emergency Incision and Drainage (I & D) failed intubation protocol [16]	1	3	4	3	3	1	1	3	3	3	5	2	2	5	2	1
75% burns with hyperkalemia and Hemoglobin (HB) 2 shock resuscitation protocol [17]	5	4	4	1	5	2	2	4	4	4	4	1	5	3	2	2
90 years with diabetes mellitus (DM), hypertension (HTN), chronic obstructive pulmonary disease (COPD), coronary artery disease (CAD), ischemic heart disease (IHD), post-coronary artery bypass grafting (CABG), dilated cardiomyopathy (DCMP), low ejection fraction of 20% (low EF 20), diabetic ketoacidosis (DKA) for emergency laparotomy concerns [18]	3	1	3	2	4	2	1	5	1	1	4	1	1	4	4	1
Uncontrolled Thyroid Stimulating Hormone (TSH) (150) for elective Lower Segment Caesarean Section (LSCS) modality of workup [19]	2	2	5	2	5	4	3	2	1	1	5	5	1	5	3	5

However, all the scenarios are carefully picked out and these situations are very demanding ones requiring medical expertise. In order to define the most complex situations one should take into consideration such characteristics as a rarity of condition, complications with surgery, potential risks, and overall complexity level for all medical issues. From the list provided, here are a few scenarios that stand out due to their extensive complexity:

Atrial Switch Surgery Prognosis

The atrial switch is a complicated cardiac surgery done mostly on patients suffering from congenital heart defects. Different outcomes are possible in such situations, depending on multiple variables - the patient’s overall health condition, other heart malformations, and the age of surgery.

Malignancy Periampullary Pancreas Post-operative Success Rate

Periampullary malignancies, including the anatomical site where bile and pancreatic ducts open into small intestines, are hard to cure. The rate of surgical success can also be affected by the stage of cancer, overall patient health, and the presence of metastasis.

Ninety Years With Multiple Chronic Conditions for Emergency Laparotomy

The management of a patient with diabetes, hypertension, chronic obstructive pulmonary disease (COPD), coronary artery disease, ischemic heart disease, post-coronary artery bypass graft (CABG) dilated cardiomyopathy low ejection fraction, and diabetic ketoacidosis poses a highly complicated case. The consequences of complications development during an emergency laparotomy in a patient with age and numerous comorbidities are much higher. *75% Burns With Hyperkalemia and Hemoglobin 2 Shock Resuscitation Protocol*

Skin burns covering 75% of the body, hyperkalemia (high potassium levels), and severe anemia with Hb being only two, are very complicated. Such a situation calls for detailed monitoring of fluid resuscitation, electrolyte balance, and associated complications such as infection and organ failure.

All these cases involve a multidisciplinary approach and consider various factors to achieve favorable patient results. Each case is not only surgical in complexity but requires intricate preoperative and postoperative care as well as comorbidity management and potential complications.

Descriptive statistics

Table 2 shows descriptive statistics for how well different AI models work. Claude AI is the most related, and Google Bard gets the lowest fullness score. The results of all tests suggest different ratings. ChatGPT's clarity and Claude AI completeness scores are more stable. Google Bard’s clarity, however, varies significantly. This table provides a quantitative comparison between four AI models - ChatGPT, Claude-AI analyzer, Google Bard, and Perplexity AI regarding accuracy, relevance, clarity, and completeness in carrying out multi-complex scenarios based on mean scores among parameter variations.

**Table 2:** Comparison of AI Models for Medical Scenarios

AI Model	Descriptive Statistics	Mean	Standard Deviation
ChatGPT	Accuracy	3.1	1.5
	Relevance	2.3	1.3
	Clarity	3.1	1.3
	Completeness	2.5	1.5
Claude AI	Accuracy	3.4	1.2
	Relevance	3.6	1.5
	Clarity	2.3	1.2
	Completeness	3.4	0.8
Google Bard	Accuracy	2.8	1.2
	Relevance	2.5	1.5
	Clarity	3.2	1.6
	Completeness	2.2	1.5
Perplexity A	Accuracy	2.3	1.3
	Relevance	3.	1.2
	Clarity	3.1	1.1
	Completeness	3.0	1.3

Statistical comparison

Table 3 shows there is no significant difference in accuracy, clarity, and completeness among the AI models (p >0.05), but there is a significant difference in relevance (p =0.038). Correlation analysis indicates a moderate positive relationship between Google Bard's accuracy and relevance (r =0.550, p =0.037), suggesting that as Google Bard's accuracy increases, its relevance tends to increase as well. However, no other correlations between accuracy and other metrics were found to be significant, indicating that, in most cases, accuracy does not predict relevance, clarity, or completeness within the models tested.

**Table 3:** Statistical Comparison of AI Tools ANOVA: analysis of variance

Parameters	F-Statistic	P-Value
ANOVA (Accuracy)	1.54	0.21
ANOVA (Relevance)	3.01	0.03
ANOVA (Clarity)	1.33	0.27
ANOVA (Completeness)	1.99	0.12
Parameters	r-Statistic	P-Value
Correlation (ChatGPT Accuracy-Relevance)	0.12	0.68
Correlation (ChatGPT Accuracy-Clarity)	-0.31	0.27
Correlation (ChatGPT Accuracy-Completeness)	-0.17	0.55
Correlation (Claude AI Accuracy-Relevance)	0.16	0.58
Correlation (Claude AI Accuracy-Clarity)	0.36	0.19
Correlation (Claude AI Accuracy-Completeness)	0.03	0.91
Correlation (Google Bard Accuracy-Relevance)	0.55	0.03
Correlation (Google Bard Accuracy-Clarity)	-0.37	0.18
Correlation (Google Bard Accuracy-Completeness)	0.17	0.54
Correlation (Perplexity AI Accuracy-Relevance)	-0.03	0.90
Correlation (Perplexity AI Accuracy-Clarity)	-0.27	0.34
Correlation (Perplexity AI Accuracy-Completeness)	-0.05	0.85

The comparison shows that Claude AI is better and has more information, while it points out different experiences with Google Bard's clear understanding. This shows that we need to choose a model carefully, using the performance numbers that are most important for medical decisions.

## Discussion

The comparative analysis of AI models reveals Claude AI's dominance in relevance and completeness, suggesting its superior ability to generate contextually pertinent and thorough responses. The consistency in ChatGPT's clarity and Claude AI's completeness, with lower standard deviations, indicates their reliability in maintaining a performance standard. Conversely, the significant variability in Google Bard's clarity highlights the potential for unpredictable user experiences, emphasizing the need for enhanced model fine-tuning. The ANOVA results, particularly the significant difference in relevance, further corroborate the distinct performance profiles of these models. Moreover, the moderate positive correlation between Google Bard's accuracy and relevance suggests a link between the correctness of information and its relevance suggests a link between the correctness of information and its applicability. However, such correlations are not uniformly observed across all models. This nuanced understanding of model-specific strengths and weaknesses is critical for informed AI selection, tailored to specific user needs and contexts, thus enhancing the practicality and effectiveness of AI in complex decision-making scenarios. The discussion section is further categorized into the following subsections:

Limitations

An important limitation is the risk of bias in scenario selection and AI training data. Further, the fixed behavior of AI answers does not accurately reflect dynamic decision-making in real-life clinical scenarios.

Ethical considerations

From an ethical standpoint, the use of AI in making healthcare decisions calls into question patient privacy, transparency on how and why such decisions are made through AI, and widening healthcare gaps due to biased machine learning datasets.

Contextual analysis

The findings highlight the need for context-based knowledge in AI applications within healthcare. Although AI yields helpful information, it should reinforce rather than supersede human judgment, especially in sophisticated health situations.

Future directions

Future research should be on longitudinal studies to measure the performance of AI through time and across different medical contexts. Moreover, developing approaches to incorporate AI with human governance in clinical practice is critical.

The research fortifies the changing role of AI in healthcare, jeopardizing thoughtful analysis and ethical aspects as well as balanced integration of AI into the clinical decision-making process.

## Conclusions

This paper presents a detailed comparison of AI language models in complex medical scenarios, pointing out evidence-based findings by applying quantitative analysis illustrating major differences between their accuracy and gold standards obtained by medical doctors. Comprehensive results showcase Claude AI’s abilities to provide more concise answers, whereas Google Bard's lower clarity shows that there are challenges in human-AI interactions. The current study is a crucial reference for understanding AI performance and its application in medicine. It underscores the importance of leveraging these findings to enhance AI technologies and adapt their use in medical settings. This approach aims to optimize the experience of medical professionals, ensuring they derive superior benefits from AI tools. The insights gained from this study are vital for guiding the development and effective utilization of AI in healthcare decision-making.
